# Advancements in aspiration catheter tip design for thrombectomy: a comprehensive patent review

**DOI:** 10.3389/fmedt.2024.1388638

**Published:** 2024-11-25

**Authors:** Vera G. Kortman, Yinte Verberne, Mostafa A. Atalla, Aimée Sakes

**Affiliations:** ^1^ Department of BioMechanical Engineering, Faculty of Mechanical Engineering, Delft University of Technology, Delft, Netherlands; ^2^ Department of Maritime and Transport Technology, Faculty of Mechanical Engineering, Delft University of Technology, Delft, Netherlands; ^3^ Department of Cognitive Robotics, Faculty of Mechanical Engineering, Delft University of Technology, Delft, Netherlands

**Keywords:** aspiration catheter, cardiovascular disease, cardiovascular intervention, minimally invasive surgery, medical device design, suction, thrombectomy, thrombus

## Abstract

Thrombus removal from the human body is facilitated through the utilization of aspiration catheters during minimally invasive thrombectomy procedures, where a pressure differential guides the targeted tissue through a flexible tubular medical instrument. In this paper, we present a patent analysis of thrombectomy aspiration catheter tip designs sourced from the EspaceNet database. Our findings reveal that enhancing the operability of aspiration catheters can be achieved by improving ease of positioning or suction capacity, whether through active or passive means. In terms of the former, both tip shape and flexibility play pivotal roles in maneuvering the distal end effectively. Variations in aspiration port characteristics, either distal-oriented or sideways-oriented, have the potential to enhance suction efficiency. In the active approach, aspects of positioning and suctioning are integrated into a single design, allowing for seamless transitions between configurations. While numerous design characteristics can coexist in a thrombectomy aspiration tip, a balance between flexibility and buckling resistance, as well as between maximizing aspiration lumen diameter and minimizing tip diameter, must be struck. This paper offers an insightful overview of existing thrombectomy aspiration tip designs, providing valuable inspiration for future innovations in this field.

## 1 Introduction

Mechanical thrombectomy is a minimally invasive medical procedure that plays a crucial role in the treatment of various vascular conditions, particularly those involving locoregionally formed vascular clots or thromboembolisms (1). This minimally invasive technique involves the introduction of special mechanical devices through catheters to remove potentially life-threatening occlusions from blood vessels, most commonly in the brain for ischemic stroke or the peripheral arteries. Thrombectomy can rapidly restore blood flow to endangered tissue, thereby minimizing the risk of long-term damage or disability (2). Within the realm of minimally invasive mechanical thrombectomy, aspiration, or suction, is one of the most widely used techniques to extract extraneous entities like vascular clots or thromboembolisms.

Aspiration catheters, also referred to as suction catheters, are one of the key instruments utilized for mechanical thrombectomy using aspiration. They are characterized by a slender suction lumen housing with an aspiration port at the distal end. In a typical aspiration procedure, the catheter's aspiration port is initially positioned in close proximity to the intended target site, followed by the application of negative pressure through a suction device affixed to the proximal end of the catheter. Consequently, thromboembolisms or liquids, situated in front of the aspiration port are conveyed through the catheter lumen, out of the body. The magnitude of the suction force exerted by an aspiration catheter is directly related to both the suction pressure applied and the surface area of the aspiration port.

When designing an aspiration catheter, a balance in stiffness and size is required to attain a properly functioning catheter. Firstly, the catheter needs to be flexible enough to traverse the tortuous curved vessels while remaining sufficiently stiff to transfer the suction force from one end to the other and prevent buckling. Secondly, a larger catheter opening is required to increase the suction power and reduce blockages, however, this makes catheter insertion more complicated. The compromise in stiffness and size differs depending on the surgical application; specifically, the size of the target vessels and their anatomy. A critical component that defines the requirement of both factors is the aspiration catheter tip. To fulfil the application-specific design requirements, different aspiration catheters with specific tip design features have been developed.

The three most commonly used thrombectomy aspiration catheters are *Export XT* from Medtronic, *Pronto V3* from Vascular Solutions, and *QuickCat* from Volcano Therapeutics (currently Philips) (3–5). Corresponding specifications of these catheters are summarized in Table 1.

**Table 1:** Specifications most commonly used thrombectomy aspiration catheters in 2018 (1–3).

Catheter name	Extraction lumen area [mm^2^]	Working length [cm]	Unique selling points
Export XT	0.937	140	Deliverability Kin resistance Aspiration power
Pronto V3	0.929	140	Deliverability Vessel wall adhesion prevention
QuickCat	0.858	145	Deliverability Extraction performance

This paper aims to provide a comprehensive overview of aspiration tip designs documented in patent literature, with a focus on improving the operability of these devices. Clear classification of existing approaches within the literature can serve as inspiration for the design of future innovations in aspiration catheter technology. Notably, the overview focuses on thrombi removal through aspiration catheters, a particularly challenging task due to the higher density of solids compared to fluids and gases. According to the described goal, the following definitions will be used within this paper.

Thrombectomy aspiration catheter: *a flexible tubular medical device for insertion into the human body that withdraws solid tissue through air pressure differential*.

Catheter tip: *the distal end of the catheter which consists of an aspiration lumen and an aspiration port*.

In the upcoming section, we present our criteria to find and classify relevant patents. Subsequently, we describe in more detail the different categories of our classification, explaining the innovative aspects of the corresponding patents within Sections 3, 4 and 5. Afterwards, we discuss our findings in Section 6 in which we compare the findings of the selected patents and the currently commercially available thrombectomy aspiration catheters, followed by a conclusion in Section 7.

## 2 Method

### 2.1 Patent search method

In order to get a comprehensive overview of the previously formulated problem definition, a patent search was executed on the worldwide version of Espacenet. The following search query was performed: (ti = “Aspiration” OR ti = “Suction”) AND ctxt = “Catheter” AND cpc = “A61” AND (claims = “Tip” OR claims = “Distal end”) AND desc = “Thromb*”. The resulting patents include either one of the synonyms “aspiration or suction” within their title and the word “catheter” in either the title, abstract or claims. This way, innovations concerning aspiration and applicability for catheters are included. Further, the cooperative patent classification (CPC) code A61 filters on patents within the category “medical or veterinary science and hygiene”. In order to ensure all patents include specifications about the tip design, one of the synonym search terms “tip or distal end” should be existent within the claims. Lastly, since it was chosen to focus on the removal of solid tissue, the truncation “thromb” should be mentioned within the description of the patent to ensure suitability for this application.

The patents incorporated into the literature overview resulting from the search term were selected based on the eligibility criteria. Primarily, the inclusion criteria necessitated that a patent focuses on the enhancement of the design of the catheter tip. This enhancement was required to be distinct and independent of any modifications made to other segments of the catheter, such as the shaft or proximal end. Additionally, patents featuring supplementary components other than the catheter itself in their main embodiment, such as a movable rod, were excluded from consideration. The exclusion criteria encompassed devices and technologies such as balloon catheters, stent catheters, distal ends equipped with fluid jets, designs involving active clot-busting mechanisms, innovations related to tip visibility, suction syringes, and embolic filters. It should be noted that only patents documented in the English language were considered for inclusion in this study.

The patent selection method is visualized in the Supplementary Materials. The search query, executed in August 2023, resulted in a list of 234 patents after which a manual selection was made. After eliminating duplicate entries (38), the initial patent pool was reduced to 196 records. Next, a careful examination of the abstracts, claims, and drawings was conducted to identify patents specifically related to the design of the tip. As a result, 124 patents were excluded from consideration. The remaining 72 patents underwent a more comprehensive evaluation, scanning the full text to determine if they met the eligibility criteria. After this thorough assessment, a final selection of 41 patents was chosen for further analysis.

### 2.2 Classification

**Figure 1:**
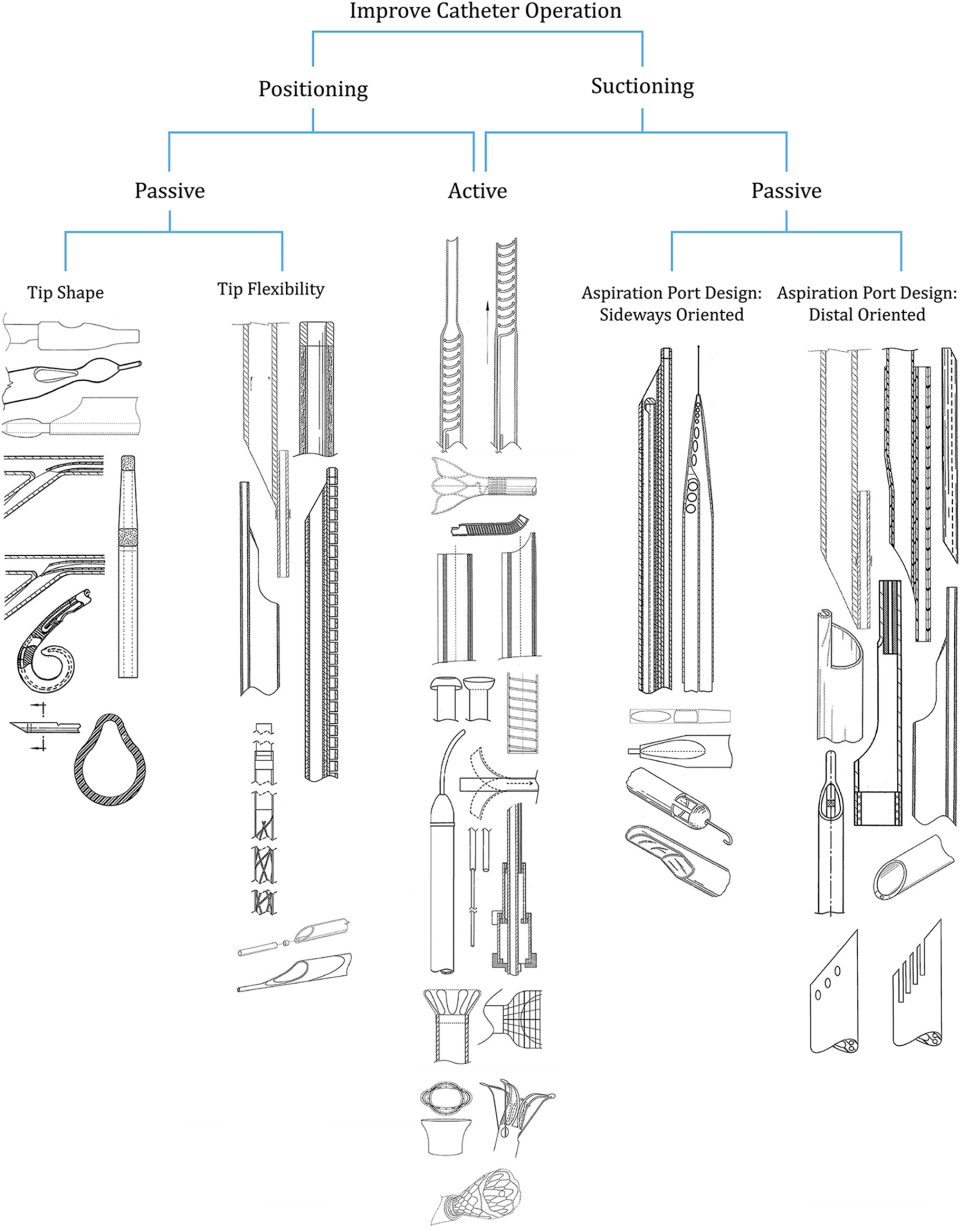
Categories of patents addressing the design of thrombectomy aspiration catheter tips.

The selected patents all aim to improve the operability of the aspiration catheter (Figure 1). This is claimed to be achieved by either (1) enhancing the ease of positioning or (2) improving the suction capacity. Both goals can be attained through a passive or active tip design. The active tip designs are beneficial for enhancing the ease of positioning as well as improving the suction capacity. For improving the positioning of the catheter passively, two subcategories were identified: (1) tip shape and (2) tip flexibility. The former addresses the shape of the distal end and the potential implementation of a guidewire to improve the ability to position the catheter in close proximity to the occlusion. The latter enhances the flexibility of the tip, subsequently improving the manoeuvrability of the catheter through the vasculature and close to the occlusion. Modifying the design of the aspiration port, and thus the cross-sectional area, directly influences the suction capacity of the catheter. A distinction can be made between patents that proposed innovative aspiration port designs that are located at the side of the catheter shaft (aspiration port design: sideways oriented) and patents proposing aspiration port designs located at the distal end (aspiration port design: distal oriented). Figure 1 gives an overview of the resulting categories, including snapshots of the corresponding 41 patents included in this review. Two patents (6, 7), occur twice within different categories.

## 3 Improve positioning – passively

Prior to the aspiration of the thromboembolism, the catheter is advanced through the body towards the target location. Within this chapter, multiple patents improving the ease of positioning through the design or flexibility of the tip will be discussed.

### 3.1 Tip shape

**Figure 2:**
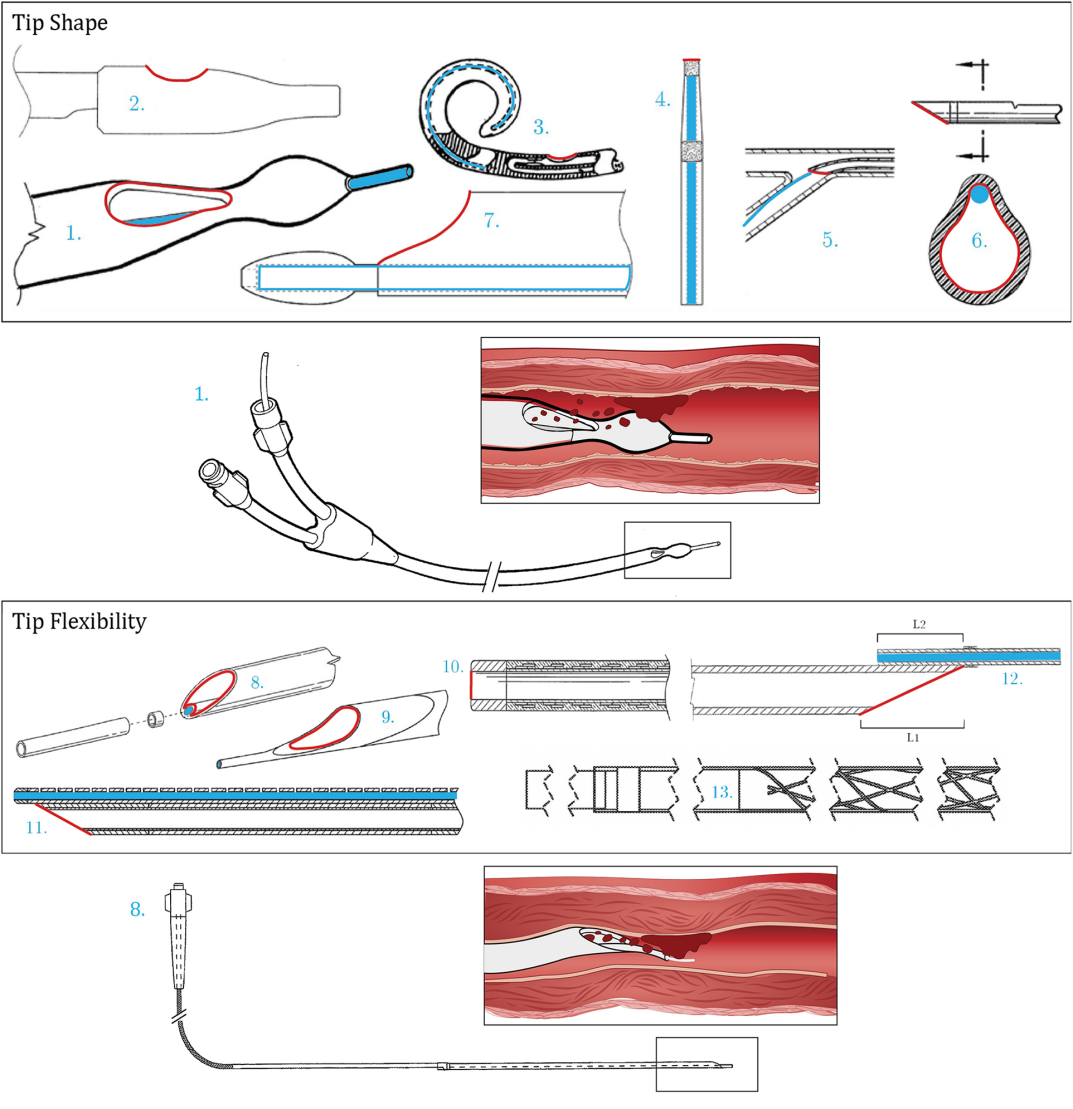
Patents concerning tip shape [1 (8), 2 (9), 3 (10), 4 (11), 5 (12), 6 (13), 7 (14)] and tip flexibility [8 (15), 9 (16), 10 (17), 11 (18), 12 (6), 13 (19)]. Color indications: Red: Aspiration port, Blue: Guidewire lumen and placement.

The patents shown in Figure 2 improve the ease of positioning due to the design of the tip of the catheter. Within Cremascoli et al. (8) (Figure 2.1), a catheter is introduced consisting of a tapered portion between the tubular body and distal end. The drop-shaped aspiration port is located behind the tip within the tapered portion of the tubular body. Ease of positioning is improved since the guidewire is centered at the tip. The tapered portion also improves the maneuverability of the distal end and the catheter operates a-traumatically due to its blunt tapered shape and softness of its tip. The patent described by Mori (9) (Figure 2.2), achieves excellent positioning over the thromboembolism through a similar configuration. Again, the aspiration port is located within the sidewall of the distal end and the guidewire is positioned co-axially at the tip. The patent emphasizes the importance of locating the aspiration port and guidewire insertion port on opposite sides with the latter longitudinally shifted in the proximal direction. Reekers (10) (Figure 2.3) introduces a preformed, curved, and pliable, pigtail-type tip improving positioning. The preformed curvature is made in the direction of the aspiration port to ensure a certain distance between the port and vessel wall to prevent potential damage to the vessel wall. The distal end of the design by Chou et al. (11) (Figure 2.4) has a tapered portion, enhancing the ease of positioning.

In the case of an obliquely cut tip, Auth et al. (12) (Figure 2.5) emphasize the importance of positioning the guidewire at the side of the aspiration port where the tip angles back. Contradictory to this, Provost-Tine et al. (13) (Figure 2.6) introduce a design in which the guidewire does not have a fixed location with respect to the aspiration port. The guidewire is located within the aspiration lumen and is free to move within this area. Both lumens are thus not separated which is claimed to be beneficial for both positioning and extraction of the catheter, as it allows for a low-profile catheter. Furthermore, the design according to Izawa et al. (14) (Figure 2.7) is characterized by the design of the protruding guidewire. The distal end of this wire is encapsulated with a tapered leading end tip member, separated from the main tube with a larger diameter than the diameter of the guidewire lumen. Due to this increase in diameter, the guidewire is bent towards the center of the distal end, minimizing interference between the suction catheter and the blood vessel during positioning.

### 3.2 Tip flexibility

Tip flexibility contributes to the bendability of the distal end and therefore improves the ease of positioning. The patents shown in Figure 2 address this matter in various ways. In order to improve the ease of positioning, Le et al. (15) (Figure 2.8) and Tanikawa et al. (16) (Figure 2.9) include an obliquely cut distal end in combination with a protruding flexible tip. This tip is preferably made from the thermoplastic elastomer Pebax and has a durometer varying between 25 and 55 and 55–72 within the former and latter, respectively. The design also reduces the risk of damaging the vessel. The hardness of the tip is decreased towards the distal end in a stepwise manner in order to prevent kinking. Within the design by Itou et al. (17) (Figure 2.10), kinking is avoided by introducing a stainless-steel reinforcing member within the soft and flexible tip. This additionally enables a thin wall-thickness of the tip. Kataishi et al. (7) (Figure 3.22) and Chou et al. (11) (Figure 2.4) include a homogeneously flexible tip. Within Kusakabe (18) (Figure 2.11), the bendability of the tip is achieved by composing the guidewire lumen of segments, each positioned side by side with an interruption in between.

**Figure 3:**
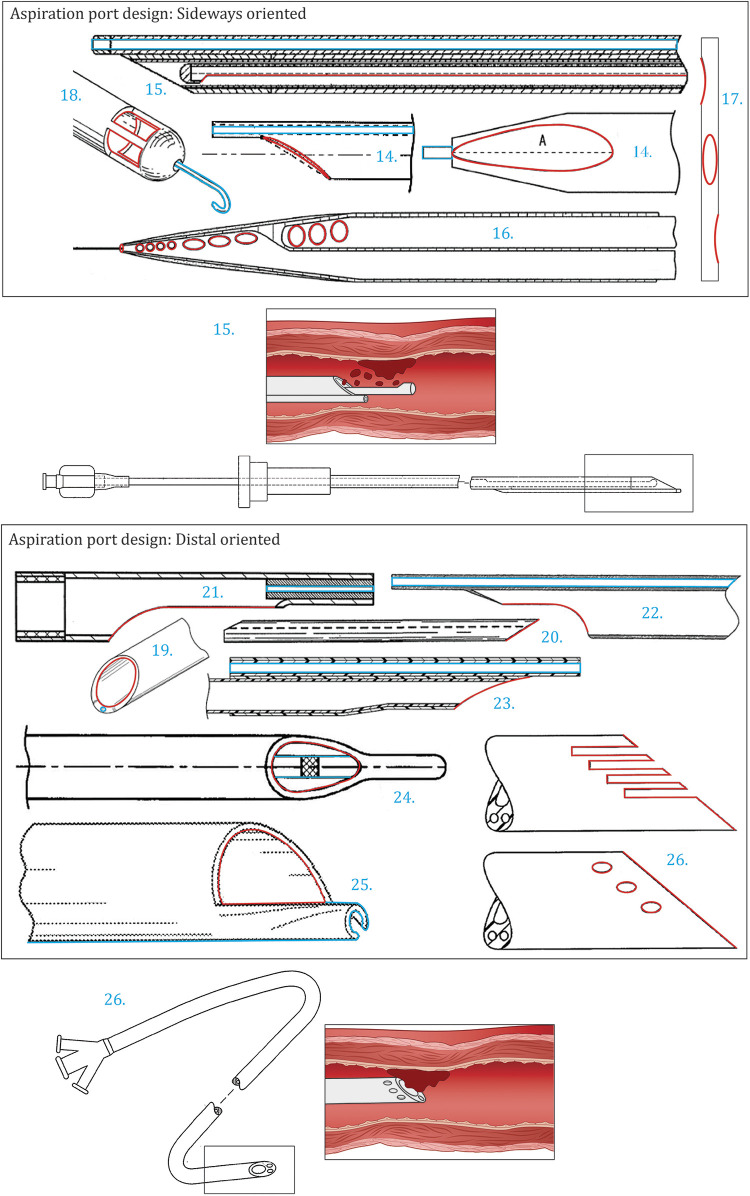
Patents concerning aspiration port design. Top: Aspiration port design: sideways oriented [14 (20), 15 (21), 16 (22), 17 (23), 18 (24)]. Bottom: Aspiration port design: distal oriented [19 (25), 20 (26), 21 (27), 22 (7), 23 (28), 24 (29), 25 (30), 26 (31)]. Color indications: Red: Aspiration port, Blue: Guidewire lumen and placement.

Within the patent described by Miki et al. (6) (Figure 2.12), the trade-off between tip flexibility and sufficient stiffness is addressed. Even though flexibility is beneficial for positioning, sufficient stiffness is required to prevent buckling and to transmit mechanical power from the proximal side to the distal end of the catheter. Attaching the aspiration lumen to a large portion of the main shaft diminishes flexibility but increases stability. According to this patent, the ideal design satisfies the following constraints: 0.5* ≤* L2/L1 and L2 - L1 *≤* 5 mm. As shown in Figure 2.12, L1 represents the horizontal distance of the obliquely cut tip and L2 represents the portion of the aspiration lumen attached to the main shaft. Lastly, it should be noted that all patents within this section mention a hydrophilic lubricative coating applied to the distal end of the catheter to further improve the ease of positioning. Corvese (19) (Figure 2.13) describes an improved catheter system that leverages PTFE tubes and liners with gaps and spaces for flexures to enable navigation through tortuous and highly constrained neurovasculature.

## 4 Improve suctioning – passively

Subsequent to positioning the catheter tip in front of the thromboembolism, the aspiration is actuated. Within this chapter, the patents including a tip design improving the suction capacity through the aspiration port design are discussed.

### 4.1 Aspiration port design: sideways oriented

The patents shown in Figure 3, all have a unique aspiration port design with varying characteristics such as shape, size, location, and the number of ports. Li et al. (20) (Figure 3.14) describe the implementation of an asymmetrical aspiration port along the virtual line A. As a result of this asymmetry, the suction pressure distribution varies over the aspiration port. The increase in suction pressure at the most proximal end of the aspiration port enhances removal. Within the design of Tanaka (32) (Figure 3.15), the aspiration port is formed as a long hole at the side of the catheter. The open area of this port is adjustable through the usage of an axially slidable inner tube. The catheter tip in this design is closed off.

Smalling (22) (Figure 3.16) claims that a plurality of aspiration ports in variable shapes and sizes enhances thrombus removal. The ports are arranged in groups along the tapered distal end of the catheter. The port size of each group gradually increases towards the proximal side of the catheter. The variability in ports results in differing aspiration vectors, increasing the effective aspiration area. Also, due to the tapered shape of the distal end, the contact between the thromboembolism and the aspiration port is enhanced. Whenever a thromboembolism is not aspirated by one of the initial ports, the thromboembolism is gradually trapped by the tip and aspirated by one of the subsequent ports. Within catheters with a constant diameter, the distance between the aspiration port and non-aspirated thromboembolism increases as the catheter advances through the vessel, therefore reducing the likelihood of removal.

Within the patents described by Wu et al. (23) (Figure 3.17) and Ruggio (24) (Figure 3.18), again a plurality of aspiration ports is incorporated within the design. Within the former, in addition to an opening at the distal end, six elliptical side holes are positioned along the tip extending to the shaft of the catheter, enhancing the removal of large thromboembolisms. The tapered shape of the distal end and rounded edges prevent tissue damage. Concerning Ruggio (24), the apertures are located along the sidewall of the hemispherical and closed catheter tip. The extending guidewire, protruding from the center of the tip, is designed to pulverize occlusions. Another advantage of multiple aspiration ports is the maintenance of functionality whenever one of the openings is blocked.

### 4.2 Aspiration port design: distal oriented

In the case of one single aspiration port at the distal end of the catheter, the configuration of the tip greatly influences the suction capacity. Within this subcategory, two approaches are discussed: (1) enhancing the suction capacity and (2) preventing disruption of the aspiration through the configuration of the distal end.

The patents shown in Figures 3.19–23 are designed to enhance the suction capacity of the catheter. Miki et al. (6) (Figure 2.12), Fojtik (25) (Figure 3.19), and Bagaoisan et al. (26) (Figure 3.20) all recommend an obliquely cut tip. By obliquely cutting the tip, the surface area of the aspiration port is increased whilst maintaining a suitable diameter of the aspiration lumen. Furthermore, this simultaneously increases the suction force and reduces the risk of clogging due to the enlarged port. Additionally, the angled opening can be beneficial for aspirating thromboembolisms positioned closer to the vessel wall and enables reduced stiffness towards the distal end. Miki et al. (6) additionally claim that the ideal length of the horizontal distance of the obliquely cut tip lies between two and ten millimeters. A smaller length is more likely to cause damage within the vessel whilst a longer length complicates removal.

The design according to Fojtik (25) optimizes the implementation of the guidewire lumen and the wall thickness of the tip. The guidewire lumen is incorporated within the walls of the aspiration lumen. The thickness of the walls is minimized in order to maximize the aspiration lumen within the given constraints. This resulted in an unevenly distributed wall thickness in which the thicker part consists of the guidewire lumen. The overall stiffness remains sufficient in order to prevent kinking.

Within the patent by Mori (27) (Figure 3.21), the distal end is not only cut obliquely but also in a concavely curved manner. The curved and rounded edges increase the cross-sectional area of the aspiration lumen and are beneficial for the positioning of the catheter within the blood vessel. The same applies to the designs according to Kataishi et al. (7) (Figure 3.22) and Li et al. (28) (Figure 3.23) in which the distal end is cut in a combination of curved and angled parts. Within the latter, the ease of manufacturing is taken into account as well.

The suction force required for thromboembolism removal can be disrupted in multiple manners. The aspiration port can be blocked by the thromboembolism or the aspiration lumen can collapse due to kinking. Both phenomena prevent airflow through the device, which is required for aspiration. The patents shown in Figures 3.24–26 focus on an approach to prevent this loss of functionality. The distal end of the catheter described by Gülcher et al. (29) (Figure 3.24) has a nose-shaped element that penetrates the thromboembolism before contacting the aspiration port. Subsequently, only fragmented pieces are aspirated, preventing blockage of the port. Comparable with the previously discussed design of Ruggio (24) (Figure 3.18), the extending guidewire is used to pulverize the occlusions. Within the patent by Consigny (30) (Figure 3.25), the distal end compromises a cutting edge, spirally tapered along the oblique tip. Through this configuration, obstacles adhered to the vessel wall are removed whilst advancing the catheter through the body. Additionally, it is possible to rotate the device in the direction of the spiral during positioning to further enhance the removal. Goff et al. (31) (Figure 3.26) introduce multiple designs of the distal end which disrupt potential sealing between the aspiration port and thromboembolism. This can be done through axial slots or additional ports complicating the complete sealing of the lumen. Within these designs, the desired effect is achieved through the passive design of the distal end. Alternatively, the patent introduces active manners or a combination of both. For example, potential sealing between the aspiration port and thromboembolism can be disrupted by introducing a vibrating wire.

There is a trade-off between maintaining sufficient flexibility of the tip of the catheter to enhance positioning and prevent damage to surrounding tissue and maintaining sufficient structural rigidity to prevent buckling through which the aspiration lumen is sealed and suctioning is hindered. Within the before-mentioned patent introduced by Gülcher et al. (29) (Figure 3.24), a stabilization wire is led through the tip, limiting the folding tendency. Alternatively, a reinforcing member within the distal end can be implemented (16, 17, 32) or a stiffness gradient can be added (16).

## 5 Improve positioning and suctioning – actively

**Figure 4:**
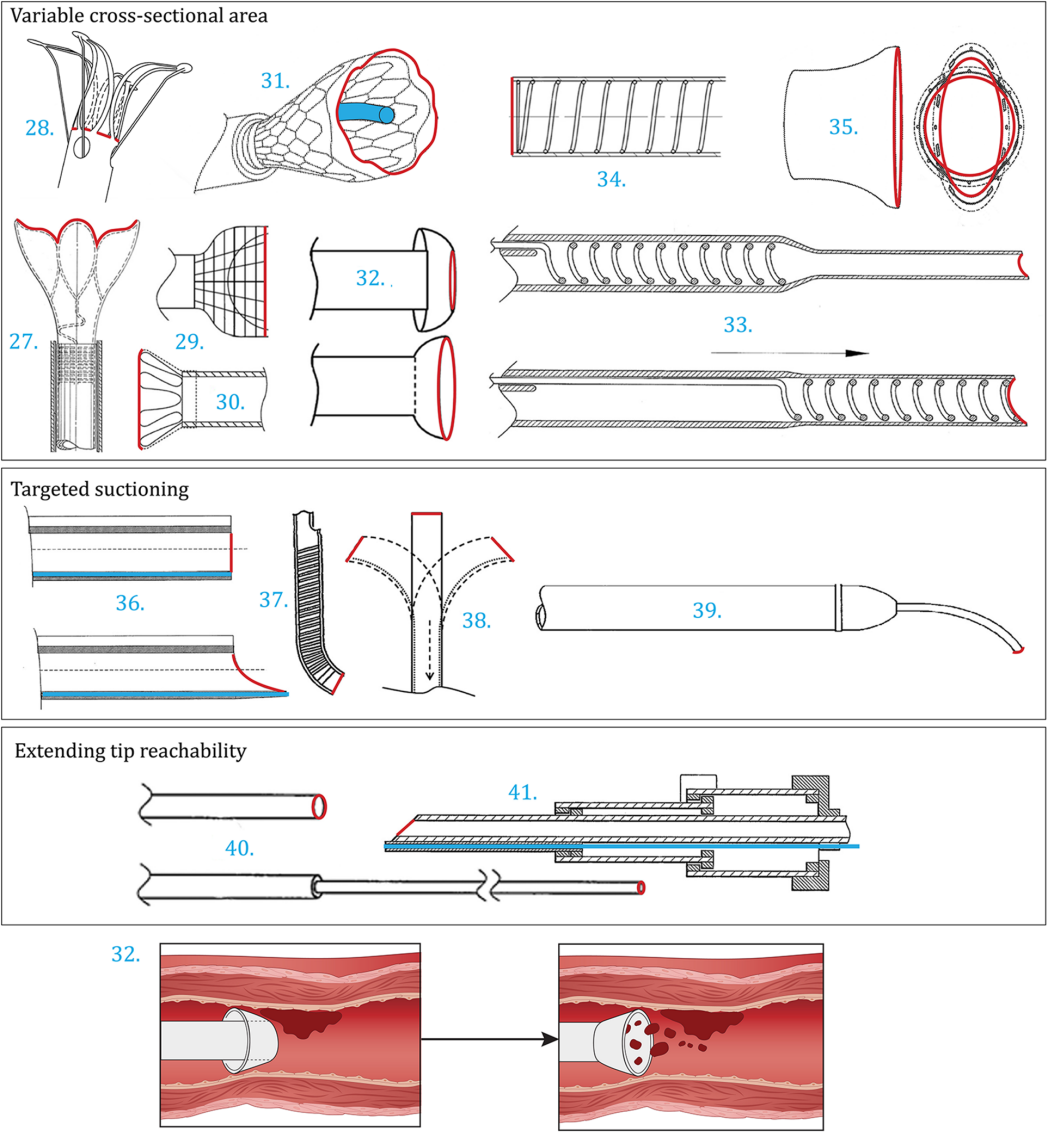
Patents improving suctioning and positioning by actively increasing the cross-sectional area of the aspiration lumen [27 (33), 28 (34), 29 (35), 30 (36), 31 (37), 32 (38), 33 (39), 34 (40), 35 (41)], by actively enabling targeted suctioning [36 (42), 37 (43), 38 (44), 39 (45)], or by actively extending the reachability of the tip [40 (46), 41 (47)]. Color indications: Red: Aspiration port, Blue: Guidewire lumen and placement.

The patents within this category focus on improving positioning or suction capabilities of the catheter tip in an active manner by switching between states, varying the cross-sectional area of the aspiration lumen, enabling more targeted suctioning, or extending the reachability of the tip, see Figure 4.

### 5.1 Variable cross-sectional area

The patents shown in Figures 4.27–35 increase the surface area of the aspiration lumen after positioning the distal end at the target location. Within Vale et al. (33) (Figure 4.27) an expandable tip is introduced. In the collapsed state, the cross-sectional area of the tip is equal to the catheter shaft, making the catheter low in profile, flexible, and thus suitable for positioning. After reaching the target location, the distal end is expanded radially, increasing the suction port diameter, and enabling the aspiration of the thromboembolism. The enlarged, funnel-shaped, port makes it possible to remove larger thromboembolisms from the blood vessel. Within this example, the expandable tip compromises a strutted framework, supplying mechanical support, encapsulated with a flexible cover. The expandable state of the cloth retrieval catheter is actuated by pulling the outer tubing backwards, also known as reversibly driven. A similar device is described by Mintz (34) (Figure 4.28) in which the expandable member includes a plurality of tensile-actuated axial prongs and/or a basket comprising struts that are configured to be expanded radially outwards in response to a tensile force applied to the pull members. Alternatively, a self-expanding structure can be introduced. Similar expandable distal ends are introduced within Li et al. (35) (Figure 4.29), Nguyen et al. (36) (Figure 4.30) and Baron et al. (37) (Figure 4.31), although slightly different in shape. Furthermore, the design described by Casey et al. (38) (Figure 4.32) utilizes the environment to switch between configurations. The tip inverts whenever retracted, creating a funnel-shaped tip with an enlarged distal end diameter compared to the transition phase.

Von Hoffmann (39) (Figure 4.33) and Li et al. (40) (Figure 4.34) both uniformly increase the diameter of the distal end prior to suctioning. Within the former, this is enabled by an axially movable support coil. The aspiration lumen is made from a stretchable material or a tubular wall that is folded within the preferred configuration. After delivery to the target location, the movable support is translated to the distal end of the catheter, subsequently enlarging the diameter of the aspiration lumen. The recommended length of the support is 5–15 cm. Within Li et al. (40), a spring component is implemented in the catheter wall which can be stretched or compressed. This way, the stiffness and diameter of the distal end can be altered simultaneously by pulling one of the two pulling members attached to the proximal or the distal end of the spring, subsequently stretching or compressing the spring, respectively. The low stiffness and small diameter state is beneficial for positioning, whilst the opposite is desired during the aspiration phase. Desai et al. (41) (Figure 4.35) describe a catheter with a customizable size and shape of the distal lumen using pull wires.

### 5.2 Targeted suctioning

Instead of enlarging the aspiration port for suctioning, the second collection of patents within Figure 4 focuses on the more targeted aspiration of the thromboembolism. During delivery, the distal ends described by Spurchise et al. (42) (Figure 4.36) and Ogle et al. (43) (Figure 4.37) are both cylindrically shaped with an orthogonal distal end. When positioned, the tip changes in shape, beneficial for aspiration. Within Spurchise et al. (42) this is done by switching between an orthogonal and oblique tip shape using a core wire. The oblique configuration enlarges the aspiration port surface area and allows bending the tip towards the thromboembolism. The distal end shown in the design described by Ogle et al. (43) consists of at least three segments, enabling a curved state in the direction of the thromboembolism for targeted removal. The distal end introduced by Laub (44) (Figure 4.38) includes a steerable tip that can be operated by the surgeon using one or more steering wires. Within Phelps et al. (45) (Figure 4.39), the tip is steerable as well. However, the diameter of this tip is sufficiently smaller, focusing on the removal of plaque. Also, rotational movement is enabled.

### 5.3 Extending tip reachability

Depending on the area of application, the diameter of the vessel can vary. For example, thromboembolism removal within the brain requires catheters with a smaller diameter when compared to removal within other parts of the body. The patents shown in Figures 4.40, 41 anticipate to these scenarios, by introducing a smaller diameter tip that can be extended to enhance the reachability of the catheter. Yang et al. (46) (Figure 4.40) and Douk et al. (47) (Figure 4.41) both introduce a catheter with a telescoping structure. Whenever constraint, the smaller diameter, low-profile, distal end can be axially extended to further advance through the vessel to the target location for removal. Within Douk et al. (47), this structure is incorporated within the catheter shaft as well. The design consists of three telescoping segments in total.

## 6 Discussion

### 6.1 Summary of main findings

In this study, we give an overview of patent literature addressing thrombectomy aspiration catheter tip designs. In total, 41 patents are incorporated within this study, presenting innovations on the distal end of the catheter without introducing add-ons. It was found that the designs can be categorized into three groups, all improving the overall operability of the catheter. Namely, by improving the ease of positioning passively (13), improving the suction capacity passively (13), or by addressing both elements simultaneously in an active manner (15). The designs introduced by Miki et al. (6) (Figure 4.12) and Kataishi et al. (7) (Figure 6.23) are incorporated twice in two categories, as shown in Figure 2. These innovations enhance both positioning and suctioning passively, by increasing tip flexibility and the effective cross-sectional area of the aspiration lumen through the distal end configuration.

Improving positioning passively includes innovations concerning tip shape (7) and tip flexibility (6). The former group mainly addresses the shape of the tip and the guidewire implementation. The flexibility of the tip can either be implemented in a stepwise manner towards the distal end or homogeneously. Within the category of patents passively improving the suction capacity of the catheter, both the sideways aspiration port design (5) and distal end aspiration port design (8) are influential factors. Concerning the former, aspiration port characteristics can vary in shape, size, location, and number of ports. The configuration of the distal end can enhance suction capacity by maximizing the surface area of the aspiration port through a non-orthogonal cut. Additionally, suction disruptions, such as sealing of the aspiration lumen and aspiration port blockage should be prevented. Within an active tip design, both goals, improving positioning and suctioning, can be achieved simultaneously by switching states. The collapsed state is low in profile and flexible, therefore maneuverable. Subsequently, the expanded state increases the cross-sectional area of the aspiration lumen, enabling more targeted suctioning of the thromboembolism, extending the reachability of the tip, and preventing kinking of the tip to enhance removal. Therefore, by implementing these key areas for improvement in aspiration catheter design, safer and more efficient thrombectomy procedures are envisioned in the future.

**Figure 5:**
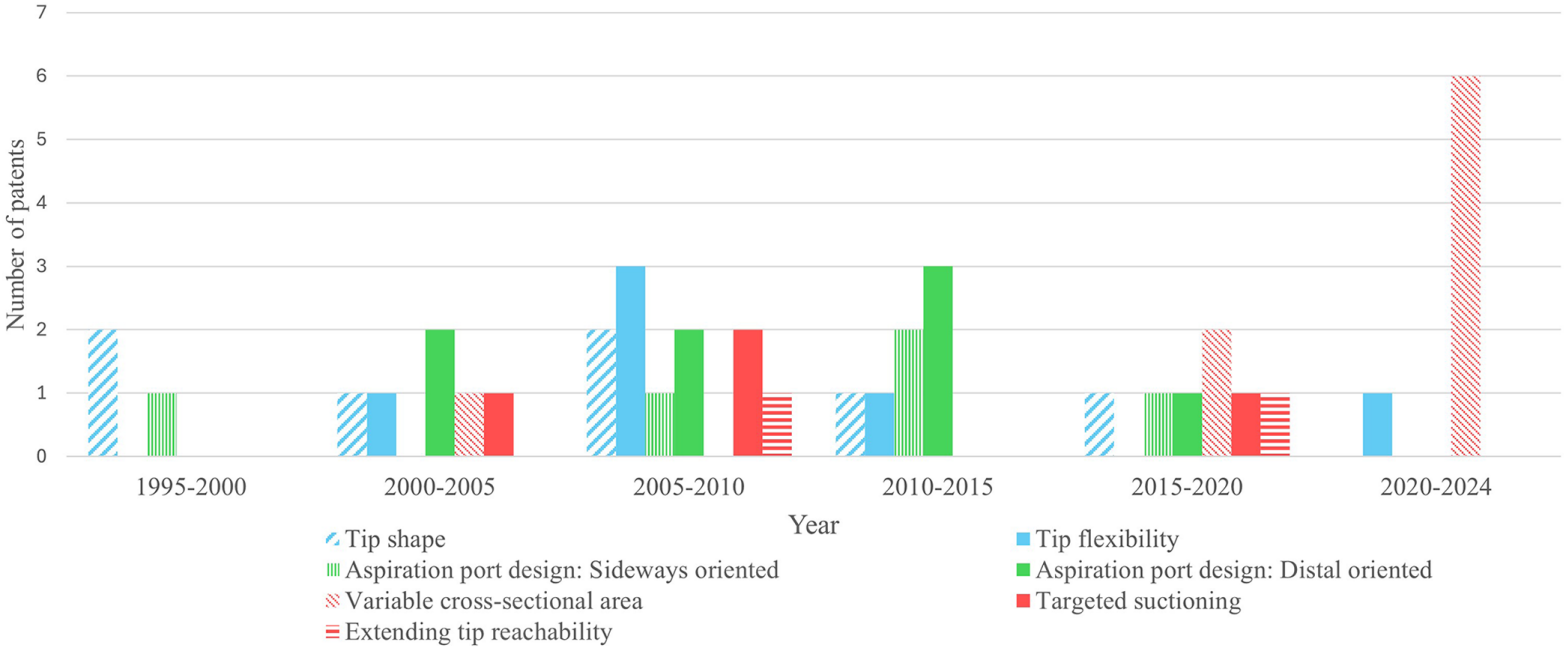
Distribution of the included patents, classified on catheter tip designs that improve the operability of the aspiration catheter by (1) passively enhancing positioning (blue), (2) passively improving suctioning (green) or (3) actively improving the positioning and suctioning (red).

The distribution of patents over the last three decades, Figure 5, shows a shift from research focused on optimizing the tip design, towards increased interest in variable cross-section catheter designs. This observation implies a trend towards improving active positioning and suctioning of catheter tips and might be fueled by recent research involving advanced actuation mechanisms using shape memory and superelastic materials like Nitinol, as well as magnetic actuation. The opposite trend is seen for patents focusing on distally orientated aspiration port designs. Furthermore, we see patents focusing on extending the tip reachability from 2010 onwards, which might allow for transferring through smaller blood vessels in the future.

#### 6.1.1 Atraumatic tip

The three discussed categories were drawn up based on the main goals addressed within the selected patents. However, many patents additionally include improvements to the catheter tip in order to prevent trauma to surrounding tissue. Three different approaches can be found in the identified patents. Firstly, by applying a coating to the distal end, friction between the catheter and vessel wall is decreased. This simultaneously makes it easier to reach the target location and minimizes harm (Approach A). Additionally, it is possible to apply the low friction coating on the inside of the aspiration lumen, to reduce the possibility of the thromboembolism sticking to the inside. Other patents mention softened or rounded tips to prevent damage (Approach B). Furthermore, hindering suctioning between the vessel wall and the aspiration port is beneficial for an atraumatic treatment (Approach C). Table 2 summarizes which improvements are implemented in the selected patents, categorized according to the three mentioned approaches. Note that design specifications may overlap in resulting improvements. For example, when the ease of positioning is improved, the risk of damage is potentially reduced as well. Therefore, only patents explicitly mentioning making the catheter less traumatic are included within the table. Experiments should be executed to evaluate the effectiveness of the elements summarized in Table 2.

**Table 2:** Patents addressing atraumatic tip design. A: reduce friction between the catheter and vessel wall, B: through the configuration of the distal end, and C: prevent suctioning of the vessel wall.

Patent	A	B	C	Specifications
(6)	X			Hydrophilic coating
(12)	X			Lubricious coating (polyvinylpyrrolidone hydrogel), the usage of an antithrombogenic material (heparin complex or hirudin), and a low friction coating on the inside of the aspiration lumen
(16)	X			Lubricious coating
(22)	X			Formed or coated with smooth or low-friction and soft material (Teflon)
(27)	X			Hydrophilic coating
(30)	X			Lubricious coating (Teflon) or hydrophobic coating (silicone lubricant dispersion PN 4097 or hydrogel)
(34)	X			Flexible material or coating
(37)	X			ePTFE (Teflon) coating
(39)	X			Lubricous coating (Paralene, Teflon, silicone rubber, or polyimidepolytetrafluoroethylene composite materials)
(8)	X	X		Blood compatible coating and soft and flexible tip with tapered portion
(17)	X	X		Hydrophilic lubricous coating and a soft and flexible tip
(35)	X	X		Lubricous coating (PTFE), hydrophilic or hydrophobic, and a reduced cross-section and compliant tip
(33)	X	X		Lubricous coating (hydrogel or flexible fluoropolymer) and a funnel-shaped tip with a soft distal ridge
(15)		X		Convex edge and a flexible tip tube
(23)		X		Tapered tip with fillet shape and a rounded structure of tip
(25)		X		Rounded and smoothed distal end
(28)		X		Curved surface of the distal end (combination of concave and convex parts)
(38)		X		Low-profile, curved tip
(40)		X		Smaller and flexible distal end
(29)			X	Pressure balancing hole opposite to aspiration hole to prevent the aspiration hole to attach to the vessel
(9)			X	Additional side hole to prevent the aspiration port to get stuck to the blood vessel wall.
(10)			X	Preformed curved tip in the direction of the aspiration port ensures distance between suction inlet and vessel
(29)			X	Pressure balancing hole opposite to aspiration hole to prevent the aspiration hole to attach to the vessel

#### 6.1.2 Guidewire implementation

**Figure 6:**
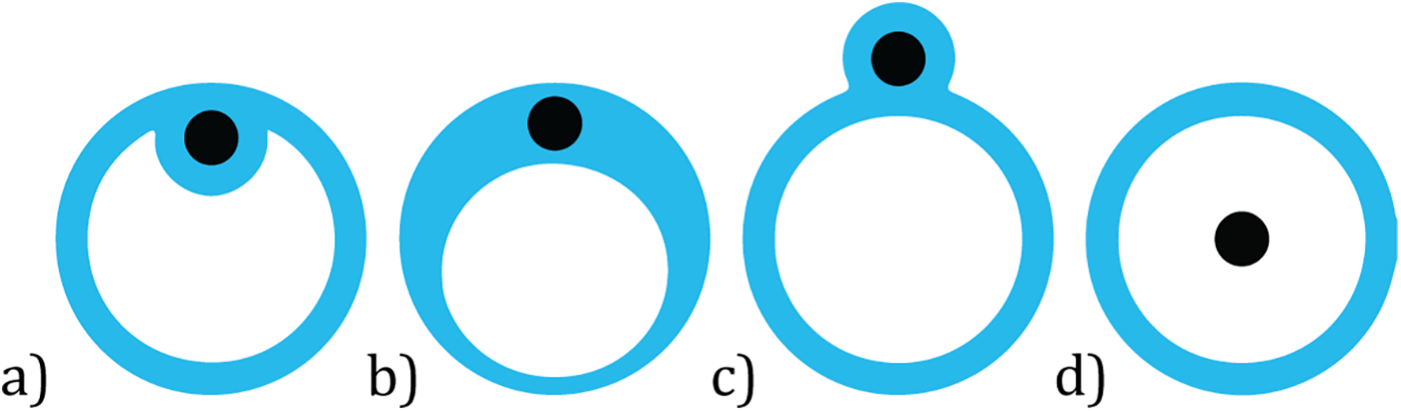
Distal end cross-sections visualizing guidewire (black dot) implementation: **(a)** fixed location within aspiration lumen, **(b)** fixed location within enlarged catheter wall, **(c)** fixed location outside aspiration lumen, **(d)** fixed or variable location within the aspiration lumen.

A guidewire can be implemented within the catheter tip. This wire is advanced to the target location first, after which the rest of the catheter body follows, potentially easing the positioning. Within Figure 6, several configurations are shown to emphasize the variability of the guidewire implementation within the selected patents. The figures visualize the distal end cross-section, in which the black dot represents the guidewire.

Within the first three configurations, the guidewires have an off-center fixed location within the distal end. Auth et al. (12) claim that in the case of an obliquely cut tip, it is beneficial for the ease of positioning to fix the guidewire on the side where the tip angles backwards. A trade-off exists between the cross-sectional area of the aspiration lumen and the overall size of the tip, since enlarging the former improves the suction capacity whilst a larger diameter of the tip complicates positioning. Also, the wall thickness and force distribution should be sufficient to prevent buckling. Within the last configuration, see Figure 6 (d), the guidewire is positioned within the aspiration lumen, at either a fixed or variable location. In the case of the former, the distal end of the tip is closed, and the guidewire protrudes through this surface. Concerning the latter, dependent on the surroundings and curvatures in the vessel wall, the guidewire can move to a suitable location within the aspiration lumen. This design is introduced within the patent by Li (20). Although, configuration (d) is the easiest to manufacture, the guidewire might hinder thromboembolism removal. Note that the implementation of the guidewire does not have to be constant along the entire length of the distal end. For example, within the design described by Ruggio (24) (Figure 3.18), configuration (a) is followed by the fixed version of configuration (d). Changing the guidewire configuration along the catheter body will allow for the optimization of the mechanical properties of the catheter but complicates manufacturing. In future, research could be conducted to determine the most optimal guidewire guidance to improve the trackability of the catheter, as well as look into innovative ways to manufacture a variable guidewire lumen along the catheter body, for example by using state-of-the-art extrusion and additive manufacturing technologies.

#### 6.1.3 State of the art

Within Table 3, the design specifications of the instruments shown in Table 1 are evaluated based on the innovations mentioned in the patent review. It is shown which design features of the included patents are present within currently clinically available aspiration catheters. It can be noted that none of the designs implements an alternative sideways oriented aspiration port design to improve the suction capacity. All three instruments consist of one single and symmetrical aperture located at the tip of the catheter. Also, all designs implement an obliquely cut tip, maximizing the cross-sectional area of the aspiration lumen, and have a lubricious hydrophilic coating. Information regarding tip flexibility was not found for all three instruments. The Pronto V3 is shown to include the most features introduced within the selected patents. In terms of real-world outcomes, Table 3 highlights the potential for enhancing current commercial aspiration catheters to better navigate towards thromboembolisms and enhance their suction capabilities without encountering issues of clogging by incorporating the strategies identified in the patent review.

**Table 3:** Similar features between the state-of-the-art thrombectomy catheters (A: export XT, B: pronto V3, and C: quickCat) and selected patents with corresponding categories.

Improve catheter operability				
Feature	Improve positioning		Improve suctioning	
	Tip design	Tip flexibility	Aspiration port design: sideways oriented	Aspiration port design: distal oriented
Fixed guidewire position at the side where the tip angles back (12)	A			
Tapered portion between body and tip (8)	B			
Centered guided (8, 9)	B, C			
Enlarged guidewire tip (14)	B			
Variable guidewire position within aspiration lumen (13)	B, C			
Lubricous hydrophilic coating (6, 12, 16, 22, 27, 30, 39)	A, B, C			
Soft tip (17)		A		
Obliquely cut aspiration lumen (6, 25, 26)				A, B, C
Prevent vessel wall adhesion (9, 29)				B

### 6.2 Clinical applications and devices

To assess the translational aspect of the patented aspiration catheter designs, we traced the companies to which patents were assigned, and we evaluated the similarities between the patented design and their commercially available instruments. Upon examining the patents, we found several examples of commercially available catheters that are based on the reviewed patent designs. One example is the *TransVascular Aspiration Catheter* (Nipro, Osaka, Japan), which features a flexible catheter tip with an oblique, concave distal end, easing catheter positioning inside the blood vessel. This tip design is described in patents by Kataishi et al. (7), Kusakabe et al. (18), and Mori et al. (27). Similarly, the *Thrombuster PRO* (Kaneka Medical Products, Tokyo, Japan) achieves tip flexibility through an oblique, asymmetrical catheter tip, as described in patents by Miki et al. (6), Li (20), and Li and Kurose (28). The *Eliminate aspiration catheter* (Terumo, Tokyo, Japan) incorporates a flexible, obliquely cut catheter tip reinforced with a braid to prevent buckling, as described by Itou and Fukuoka (17). The *Tensing 7 delivery catheter* (Route 92 Medical, San Mateo, CA) features a tapered, atraumatic tip that facilitates positioning within the blood vessel, as described in the patent by Chou et al. (11). Additionally, the *Helo PE thrombectomy catheter* (Endovascular Engineering, Menlo Park, CA) embeds a self-expanding catheter tip that ensures a wide opening for large clot removal, as described by Baron et al. (37). The safety and feasibility of this catheter design are currently evaluated in a clinical trial (48).

A common trend observed in the patents was the use of polymeric materials like PEBAX, which allows for varying hardness levels in specific areas of the shaft (13, 15, 16). In particular, one patent (16) detailed a PEBAX catheter shaft with a Shore hardness ranging from 63D to 72D at the proximal end, tapering to a Shore hardness of 40D to 55D at the distal tip. Another patent (15) described a flexible tip made from a softer 25-44D PEBAX. Additionally, several patents highlighted the advantages of incorporating a radiopaque material at the distal tip of the aspiration catheter (8, 13, 17, 38) or applying a coating with an anti-thrombogenic agent (12).

It should be noted that the patents often do not specify particular application areas or target regions. Furthermore, the overall functioning of the catheter is the sum of all parts of the device, including the shaft design and handle. However, one can infer the intended working environments of the patented catheters by looking at their tip and shaft design. Some catheters are optimized for navigating narrowed and tortuous portions of blood vessels during peripheral interventions, such as the saphenous veins (8) or the carotid and cerebral arteries (38), while others are designed for use in larger blood vessels, such as the pulmonary artery. The former category emphasizes methods to enhance tip flexibility and foldability to reach smaller blood vessels and target thromboembolisms locally (8, 17, 38), whereas the latter focuses on precise tip positioning within larger vessels (9, 10). It must also be noted that the removal of chronic or acute thromboembolisms poses different challenges, with the former being more resistant to removal. For example, in a study of Madjidyar et al. *(*49), it was found that the thrombus composition can affect the efficacy of thrombectomy. In their study, they were unable to aspirate fibrin-rich clots using the *ADAPT catheter* (SOFIA 5F, Microvention, USA), whereas the removal of “softer” erythrocyte-rich clots was also unsuccessful in one third of the cases. Even though the authors do not offer an explanation as to why the clots could not be aspirated, we hypothesize that an insufficient pressure differential and the clot composition, with strong connections to the blood vessel wall, are the main contributors. Therefore, using an aspiration port with an increased suction diameter in these cases might be beneficial. Additionally, optimal catheter design depends on the type of aspiration technique employed. Aspiration catheters can either remove the thromboembolisms through the catheter via continuous flow or trap it at the tip using vacuum force. The latter benefits from strong vacuum forces that dislodge and remove the thromboembolisms effectively. Larger diameter catheters, like those used for large vessel occlusions in cerebral thrombectomy, or those with tip designs that enhance suction are advantageous. The latter includes sideways-positioned aspiration ports (20, 22–24, 32), multiple aspiration ports (22–24) or tips with a variable cross-sectional area (33–41). These designs allow for greater vacuum forces using similar vacuum pressures. An increased vacuum pressure would require higher kink resistance by, for example, reinforcing the shaft with a braid (17). For peripheral applications with continuous flow aspiration catheters, flexibility is key to navigating tortuous vessels. These catheters might embed steerable tips (42–45), tapered tips for improved manoeuvrability (8, 9, 11) or a gradually lower durometer towards their distal end (16). Gradually adapting the durometer lowers the risk of buckling over the length of the catheter. Additionally, an extended guidewire can be used to break up occlusions before suction (24).

### 6.3 Limitations

The selected patents within this study are gathered from one database only, namely the worldwide version of Espacenet. Potentially, the results can be expanded by making use of additional databases. Specific eligibility criteria were drawn up to select a demarcated number of patents. Due to the search query, only patents mentioning the truncation “thromb” within the description were selected. Innovations mentioning this aspect only elsewhere or concerning the aspiration of other solid tissues might have been unjustly excluded. Furthermore, since the study focuses on the distal end of the catheter, improvements made in the shaft or proximal end are not incorporated in this review, although these enhancements might prove crucial for overall device operability. Additionally, some patents simultaneously address multiple design goals. In these cases, the main goal was leading for the development of the categories. Potentially, the categorization can be expanded by including the sub-goals. Finally, to overcome current limitations in aspiration catheter design, it is a necessity to establish close collaborations between the biomedical engineers designing the innovative catheters and clinicians that will utilize these devices to ensure easy integration and adoption of the improved technology into the interventions.

### 6.4 Recommendations for future research

This study gives an overview of thrombectomy aspiration catheter tip design variables, structured in different categories based on the patent design goal. This study can form the basis for future development in aspiration catheter tip designs with improved operability. For this purpose, additional research is required to assess contradictory design specifications or variables that cannot be combined. Even though not specifically described as a category, it is of importance to develop an atraumatic tip to ensure safety during navigation and operation of the catheters. In future, we envision aspiration catheters to utilize active positioning, using magnetic- or wire-actuation, to optimize efficiency and safety, with an increase in aspiration lumen size, using shape memory and superelastic materials such as Nitinol, to prevent kinking and improve thromboembolism dislodgement. We also envision additional means of thromboembolism removal to be used as an add on to aspiration, such as the wasp-inspired transport means (50, 51).

## 7 Conclusion

In this study, we give a comprehensive overview of thrombectomy aspiration catheter tip designs available in patent literature. Overall, 41 patents were included and divided over five subcategories. It was found that the overall operability of the aspiration catheters can be improved by enhancing the ease of positioning or the suction capacity, either actively or passively. Whenever the active approach is chosen, both functionalities can be addressed simultaneously by switching states, which is advantageous for an efficient and safe procedure. The overview given within this paper might serve as a source of inspiration for future innovations concerning thrombectomy aspiration catheter tip designs.
