# Supplementary figures and images for: Advancements in aspiration catheter tip design for thrombectomy: a comprehensive patent review
Source: Front Med Technol. 2024 Nov 25;6:1388638. doi: 10.3389/fmedt.2024.1388638 (PMC11625537; doi:10.3389/fmedt.2024.1388638)

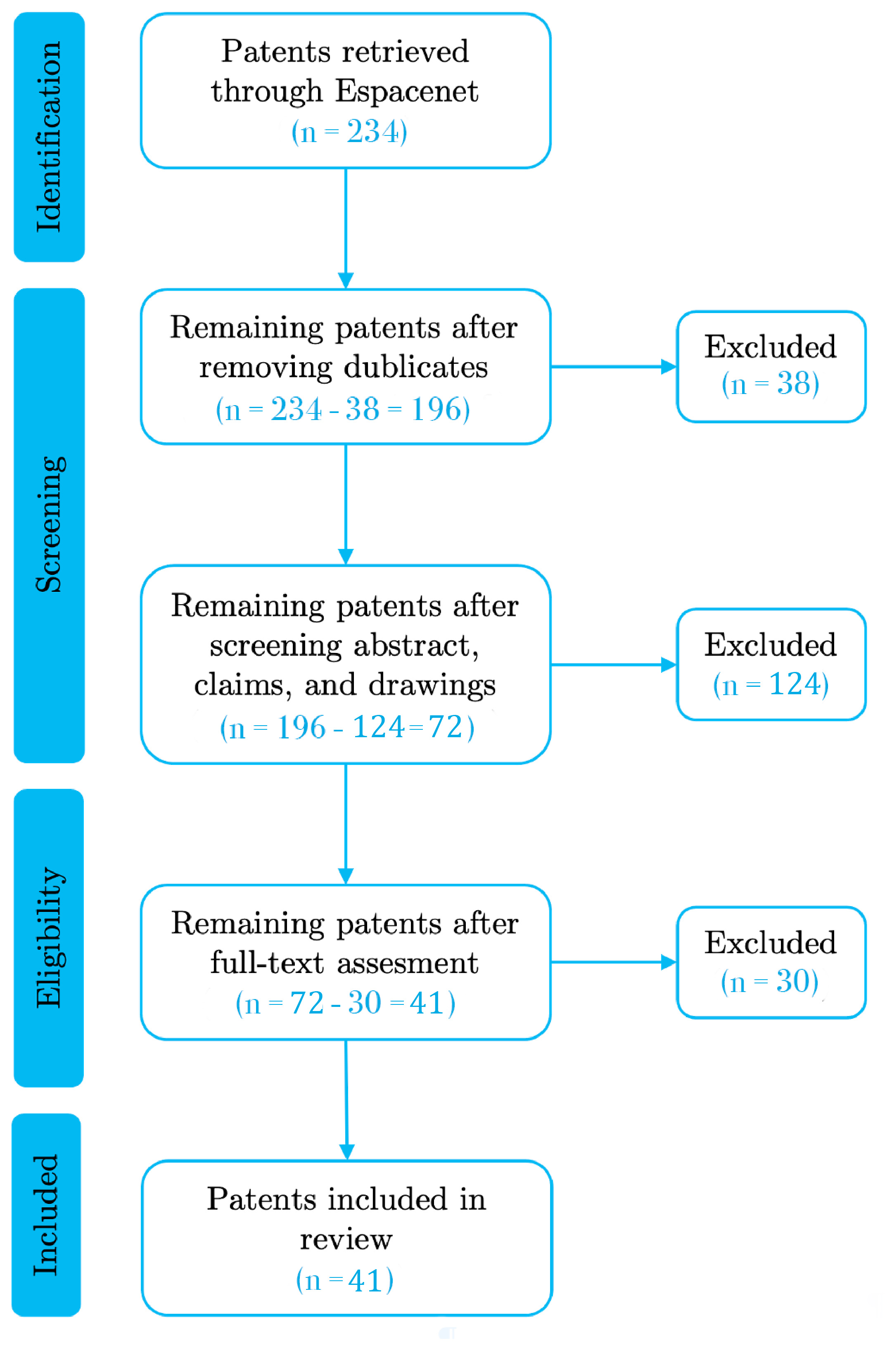

Supplement: Supplementary file 1 [file Image1.jpeg]
